# Gait Analysis After Anterior Cruciate Ligament Surgery Comparing Primary Repair and Reconstruction Techniques

**DOI:** 10.3390/jcm14145026

**Published:** 2025-07-16

**Authors:** Filip Hušek, Jiří Vitvar, Roman Mizera, Zdeněk Horák, Lukáš Čapek

**Affiliations:** 1 Department of Orthopedics and Trauma, Regional Hospital, 460 01 Liberec, Czech Republic; 2 3rd Faculty of Medicine, Charles University, Prague 10, 100 00 Prague, Czech Republic; 3 Department of Clinical Biomechanics, Regional Hospital Liberec, 460 01 Liberec, Czech Republic; 4 Department of Medical Biophysics, Faculty of Medicine in Hradec Kralove, 500 03 Hradec Kralove, Czech Republic; 5 College Polytechnics Jihlava, 586 01 Jihlava, Czech Republic

**Keywords:** ACL, proximal rupture, primary repair, InternalBrace, gait analysis

## Abstract

**Background**: ACL graft reconstruction is considered the gold standard for ACL injury treatment. Recently developed primary repair techniques such as InternalBrace ligament augmentation (Arthrex©) look like promising alternatives. The aim of our study is to compare functional results of two different surgical techniques using a gait analysis. **Methods**: A total of 42 patients who underwent surgical treatment for ACL rupture were included in this study. The first group was represented by patients who were surgically treated with ACL reconstruction. The second group included patients with acute ACL injury, who underwent primary repair with InternalBrace augmentation. Gait data were measured in the Human Motion Analysis Lab at our institution. The time interval for data collection was 6 weeks after surgery and 6 months after surgery. **Results**: There was no significant improvement in maximal and peak flexion for both group 1 and group 2 in the 6-week and 6-month intervals. Also, no significant improvement of maximal extension was found in group 1. In contrast, the study showed a reduction in maximal extension for group 2 in the 6-week and 6-month intervals. When comparing peak extension for the graft or InternalBrace techniques, no significant difference was found between both groups in the 6-week evaluation. However, results differed significantly in the 6-month evaluation. **Conclusions**: Considering the faster gain of extension, less invasiveness of the procedure, and shorter operating time, primary repair with InternalBrace augmentation seems to be a suitable option for treatment of proximal avulsions and Sherman I ACL ruptures.

## 1. Introduction

The anterior cruciate ligament (ACL) is one of the most frequently injured anatomical structures in a knee injury. ACL rupture leads to anteroposterior and rotational instability and secondary osteoarthritis of a knee joint. The timing and type of surgery are still a matter of conjecture among experts. Magnetic resonance imaging (MRI) is an indispensable diagnostic modality for the examination process of this injury. ACL ruptures were first classified by Sherman et al. [1]. Van der List et al. compared the classification with MRI results and modified the original Sherman classification for clinical use [2]. Based on these results, a surgeon can exactly evaluate the rupture type, timing, and type of surgery. ACL graft reconstruction is considered a gold standard [3,4]. In the last decade, the effort to repair the native ligament has increased again. It is assumed that primary repair surgeries lead to the preservation of proprioception and correct knee joint kinematics, which could prevent the development of secondary osteoarthritis [5].

Historically, research has been aimed at the impact of anterior cruciate ligament (ACL) rupture on gait biomechanics. The authors reported various functional patterns over time following surgery [6,7,8,9,10,11,12,13]. Mostly, a reduction in range of motion was found after ACL surgery, such as when ACL reconstruction exhibited reduced initial knee flexion angles during stair descent or reduced amplitudes of knee flexion-extension-adduction and hip adduction in the afflicted limb 1.5 years following ACL surgery. Nevertheless, at a subsequent time, usually the authors discovered no abnormalities compared to the contralateral limb. From this point of view, ACL graft reconstruction seems to be the gold standard. The question arises with newly developed primary repair techniques, specifically primary repair with InternalBrace (Arthrex, Naples, FL, USA) ligament augmentation, which looks like a promising alternative to graft reconstruction [5]. The aim of our study is to compare functional results of two different surgical techniques by using a gait analysis: graft reconstruction with semitendinosus tendon and primary repair with InternalBrace ligament augmentation. The primary hypothesis is that there is no difference in range of motion (ROM) between these two surgical approaches.

## 2. Materials and Methods

### 2.1. Patient Selection

The study was conducted under ethical approval of the local ethics committee (Regional Hospital Liberec, Czech Republic) and in accordance with the Human Tissue Act of 2004. The study was performed on data from 42 patients who underwent surgical treatment for ACL rupture in the years 2023–2024 at the Regional Hospital Liberec. The following criteria were considered: sex, age, comorbidities, classification according to Sherman and modified Van der List [2], timing of surgery, and BMI. Patients with ACL injury were divided into two groups according to the type of rupture on MRI scan and the timing of surgery. There was no random division of patients because of different surgery timing. Surgery is indicated acutely within the first 4 weeks after injury and only in patients with proximal ACL tears. ACL reconstruction is usually performed only when good range of motion is achieved, i.e., at least 6 weeks after the injury. The first group (*n* = 25) was represented by patients with subacute or chronic anteroposterior instability (i.e., ≥6 weeks after injury), who were surgically treated with ACL reconstruction using the all-inside semitendinosus graft technique with a mean age of 34.5 ± 9.2 years and a mean BMI of 25.7 ± 2.5. The second group (*n* = 17) consisted of patients with acute ACL injury. All patients were initially examined with MRI. Only those with proximal avulsions or ruptures involving the proximal 25% (Sherman I and II) and with good tissue quality were indicated for acute arthroscopy within the first 4 weeks after injury. The mean age of this group was 39.9 ± 10.4 years, and the mean BMI was 24.6 ± 2.8. Primary repair with InternalBrace augmentation (Arthrex, Naples, FL, USA) was performed in these cases. Radiological and clinical follow-up intervals were at 6 weeks and 3, 6, and 12 months after surgery.

### 2.2. Surgical Technique

**Figure 1:**
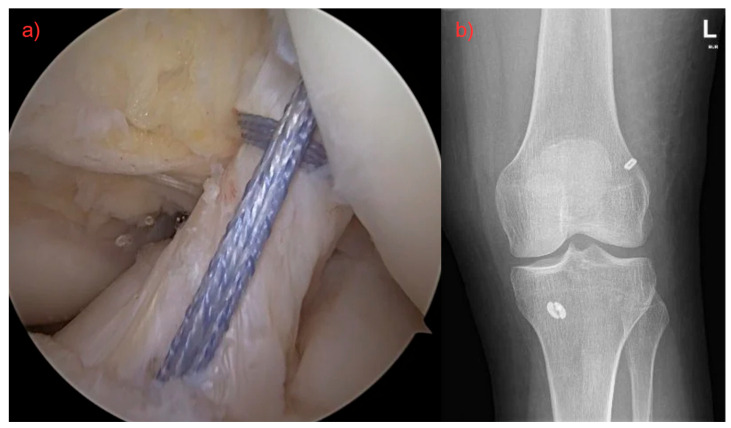
(**a**) Surgery of ruptured ligament by using FiberLink (Arthrex©), both fibers were pulled through the femoral canal, tightened, and fixed with an anchor. Final result with definitive ACL restoration. (**b**) Postoperative X-ray.

The patient was placed in supine position for both surgical techniques included in this study—primary repair and ACL graft reconstruction. Standard leg preparation and cover-up were carried out. Arthroscopic diagnosis through standard anterolateral and anteromedial portals was performed. Associated injuries (e.g., meniscal tears, osteochondral defects, etc.) were treated first, followed by ACL surgery, and everything was performed at one time. ACL reconstructions were performed using hamstring autografts. The all-inside technique was used in all cases, with an average length of surgery of 62 ± 4 min. The primary repair technique with InternalBrace augmentation was exclusively used for all patients in group 2. The distal LCA stump is stretched using a grasper to assess its length and decide whether it is suitable for primary repair of the ligament. A tibial guide is inserted through the AM port and placed centrally at the distal LCA insertion site. A 2.4 mm diameter wire is inserted through the guide, and if the position is satisfactory, the tibial canal is drilled along the wire with a 4.5 mm diameter cannulated drill. Using a wire with a loop, an auxiliary fiber is passed through the tibial canal and the base of the LCA, which is temporarily brought out of the joint through the AM port. Then, the distal LCA stump is sutured using FibreLink (Arthrex, Naples, FL, USA). The femoral canal is drilled in flexion of the knee joint with a 4 mm diameter wire. FiberLink with a stitched ligament and the TightRope implant with FiberTape (both Arthrex, Naples, FL, USA) is then pulled through the outer cortex. By tightening the individual ends of the fibers proximally alternately, the staple is fixed from the TightRope implant to the lateral cortex of the femur. Then, the FiberLink is knotted with a stitched ligament in extension to the femoral part of the TightRope implant. The suture is tightened and the ACL is toned so that full range of motion in the joint is maintained while achieving anteroposterior stability (Figure 1). The average length of surgery was 43 ± 3 min.

### 2.3. Gait Analysis Protocol

Gait analysis evaluation is the primary postoperative tool for measuring the functional outcome of this surgery. It offers objective and comparable biomechanical data related to walking. Gait data were measured in the Human Motion Analysis Lab at our institution. The time interval for data collection was 6 weeks and 6 months after surgery. At each follow-up, instrumented motion analysis was performed using twelve infrared cameras (Qualisys, Göteborg, Sweden) with a sampling rate of 200 Hz. Biomechanical analysis was performed using the IOR model [14] and Qualisys clinical gait module. For every follow-up measurement, all participants were asked to walk at a self-selected speed before measurement. A total of four steps were analyzed for the affected leg, with every step containing a complete recording from heel strike to toe off. Overall, five gait trials were performed for each patient. Maximum values of the kinematic parameters were analyzed for specific gait phases obtained from ipsilateral and contralateral foot events for the operated leg. The knee flexion and extension values were selected as a key clinically relevant parameters needed to evaluate the functional outcome of the surgery. Additional, less clinically relevant parameters such as speed, cadence, and step width were also analyzed.

### 2.4. Statistical Analysis

Data were analyzed using statistical software SPSS version 21 (IBM Corp., NewYork, NY, USA). The statistical significance of the mean difference in those two subgroups was tested with the non-paired Mann–Whitney test given the low number of subgroups. The significance level for these tests was set to *p* < 0.05. The effect size was evaluated using Cohen’s *d*, which quantifies the magnitude of the difference between groups.

## 3. Results

**Figure 2:**
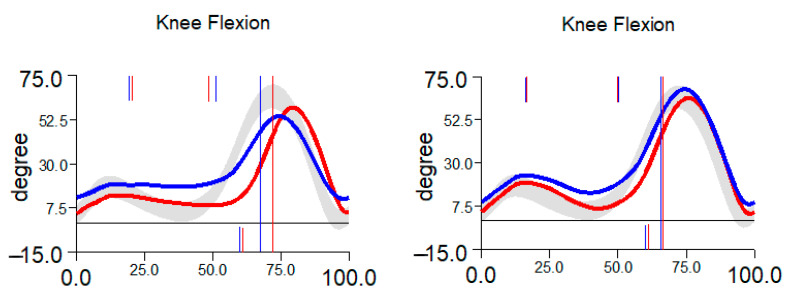
Typical restoration of knee flexion between 6 weeks (**left**) and 6 months (**right**). Blue curve represents right limb, red curve represents left limb.

There is no statistical difference between the tested groups in age (*p* = 0.09) and BMI (*p* = 0.227). The power analysis (Cohen’s coefficient) showed small-moderate and moderate-large size effects. The main factors when considering the functional results of ACL repair by the orthopedic surgeon comprise the restoration of ROM of the injured knee after surgery. Mainly the knee flexion and extension are examined 6 weeks and 6 months after surgery. The summary of results is shown in Table 1, and the representation of gait graphs is in Figure 2.

**Figure 3:**
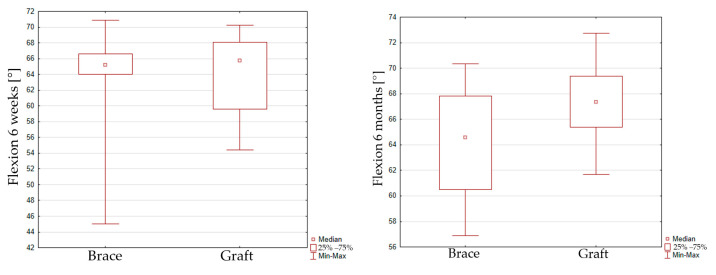
Box plot graph showing peak flexion for the graft and InternalBrace techniques in the 6-week (**left**) inspection and the 6-month (**right**) inspection.

The peak flexion at 70% of the gait cycle reaches 63.8 ± 5.4 [°] in the 6-week interval and 67 ± 2.9 [°] in the 6-month interval for group 1. The peak flexion value reaches 63.3 ± 7.2 [°] in the 6-week interval and 64.4 ± 3.9 [°] in the 6-month interval for group 2. No significant improvement of maximal flexion was seen for group 1 (*p* = 0.1066) and group 2 (*p* = 0.2167) in the 6-week and 6-month intervals. When comparing peak flexion for the graft or InternalBrace techniques, it can be seen that there is also no significant difference between both groups in the 6-week inspection (*p* = 0.8768) and the 6-month inspection (*p* = 0.0619), Figure 3.

**Figure 4:**
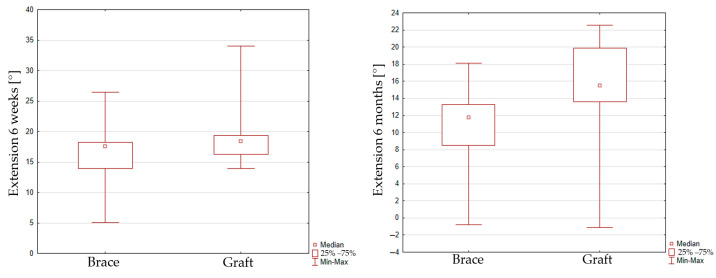
Box plot graph showing peak extension for the graft and InternalBrace techniques in 6 weeks (**left**) inspection and 6 months (**right**) inspection.

The peak extension at 40% of the gait cycle reaches 18.2 ± 5.4 [°] in the 6-week interval and 15.1 ± 5.6 [°] in the 6-month interval for group 1. The peak extension value reaches 16.9 ± 5.9 [°] in the 6-week interval and 10.6 ± 4.8 [°] in the 6-month interval for group 2. No significant improvement of maximal extension was seen for group 1 (*p* = 0.0604). In contrast, a reduction in maximal extension for group 2 (*p* = 0.0051) in the 6-week and 6-month intervals was observed. When comparing peak extension for the graft and InternalBrace techniques, it can be seen that there is also no significant difference between both groups in the 6-week inspection (*p* = 0.2776), but significant difference was found for the 6-month inspection (*p* = 0.0066), Figure 4.

There was no statistical difference between walking speed and step length between groups 1 and 2. The average walking speed for patients of group 1 changed from 1.1 ± 0.3 to 1.2 ± 0.2 m/s with step length from 63.1 ± 10.8 to 68.3 ± 5.1 cm. The average walking speed for patients of group 2 changed from 1.1 ± 0.2 to 1.2 ± 0.1 m/s with step length from 60.8 ± 6.7 to 65.1 ± 4.1 cm.

## 4. Discussion

In clinical gait assessment, both a person’s ability to walk and “how” the individual walks after the surgery are highly relevant and belong to one of the functional outcomes of the surgery. The kinematics of patients after surgery of the anterior cruciate ligament by graft reconstruction have been of great interest to various authors [15,16,17,18,19,20,21,22,23,24]. Abnormal gait patterns are commonly reported to last for months or even years after ACL reconstruction. The most consistent findings following ACL reconstruction are changes in knee flexion angles during walking. Patients with ACL reconstruction in the early postoperative period (<6 months) showed increased knee flexion angles when walking compared to undamaged contralateral knees and healthy participants. These changes are associated with a variety of conditions, including joint swelling, discomfort, and muscular weakening. However, 6 months after ACL reconstruction, it was discovered that ACL reconstruction patients may walk with lower knee flexion angles than contralateral knees or healthy people.

Data in this study showed equivalent outcomes of knee flexion between primary repair and ACL reconstruction techniques. There was no significant difference between these two groups either after 6 weeks or after 6 months, and the results of both groups were comparable. Nevertheless, disparities in knee extension measurements were found. Patients after ACL reconstruction showed minimal improvement in extension between the first inspection in 6 weeks and the second one in 6 months. On the other hand, significant improvement of extension was found in the second group after primary repair surgery. When comparing peak extension for the graft or InternalBrace techniques, it was found that there is also no significant difference between both groups in the 6-week inspection, but significant difference was found for the 6-month inspection. Thus, our hypothesis was not proved, and it seems that the InternalBrace technique provides better knee extension results in 6-month perspectives. However, the clinical outcome of this study is significant and confirmed by the results of gait analysis. What is more, in accordance with available literature, the results of our study may also point to a common complication of the ACL reconstruction procedure, which is extension deficit [25]. Understanding how injury impacts knee joint biomechanics is critical for building knee assistive devices and optimizing rehabilitation exercise programs. Recent developments in biomechanical modeling have emphasized the need for more precise, dynamic, and individualized assessment tools to understand ligament fatigue failure and long-term joint mechanics. For instance, Xu et al. [26] introduced a data-driven deep learning framework that integrates subject-specific musculoskeletal modeling, continuum damage mechanics, and real-time sensor data to predict ligament fatigue failure risks under realistic movement conditions. This approach overcomes the limitations of static biomechanical testing and provides a minimally invasive method to dynamically monitor ligament loading and damage evolution. While our study primarily focused on functional outcomes and kinematic differences between ACL reconstruction and primary repair techniques, such as InternalBrace, future research could benefit from integrating these advanced modeling tools to better assess cumulative ligament stress and predict long-term joint health post-surgery. However, existing understanding does not meet the needs of a designer and a rehabilitative physician. And it is difficult to find all studies that can comprehensively investigate all aspects of knee biomechanics [27,28,29,30].

Some limitations of the current studies must be noted. First, there is inherent variability in human mobility and gait is not carried out in the same way each time [31,32]. Movement patterns linked to functional tasks naturally exhibit both intra- and inter-subject heterogeneity, and thus there is some variability in the measurements [33,34]. Another limitation of this study is the short-term follow-up. Patients were divided according to their MRI scan and the type of rupture. Therefore, the timing of surgery differed according to the chosen type of surgery. The results support our hypothesis on the early recovery benefits of primary repair techniques in appropriately selected patients. Nevertheless, the study may lack sufficient power to detect differences in flexion, and a larger study is needed.

The InternalBrace technique represents a relatively new and evolving approach in the surgical management of ACL injuries. Unlike traditional ACL reconstruction, which has been standardized and refined over many years with well-documented long-term outcomes, the InternalBrace method is still under active development and optimization. Recognizing these differences is crucial when interpreting comparative studies, as the newer InternalBrace technique offers a potentially valuable alternative but requires further investigation to establish its long-term efficacy and best practice parameters.

## 5. Conclusions

As a developing approach in ACL injury treatment, the InternalBrace technique offers a promising alternative to conventional reconstruction methods. Considering the faster gain of extension compared to ACL reconstructions found in this study, the primary repair with InternalBrace augmentation seems to be a suitable option for proximal avulsions and Sherman I ACL rupture treatment. Confirmation of these results will require randomized trials with larger cohorts and integrated biomechanical evaluation of knee function across the surgical timeline.

## Figures and Tables

**Table 1:** Peak values of measured flexion and extension by using the graft and InternalBrace techniques.

	6 Weeks		6 Months	
	Flexion [°]	Extension [°]	Flexion [°]	Extension [°]
Brace	63.3 ± 7.2	16.9 ± 5.9	64.4 ± 3.9	10.6 ± 4.8
Graft	63.8 ± 5.4	18.2 ± 5.4	67 ± 2.9	15.1 ± 5.6

## Data Availability

No new data were created or analyzed in this study.
